# The Outcome of under 10 mm Single-Incision Surgery Using a Non-Specialized Volar Plate in Distal Radius Fractures: A Retrospective Comparative Study

**DOI:** 10.3390/jcm12247670

**Published:** 2023-12-14

**Authors:** Chang-Yu Huang, Chia-Che Lee, Chih-Wei Chen, Ming-Hsiao Hu, Kuan-Wen Wu, Ting-Ming Wang, Jyh-Horng Wang, Tzu-Hao Tseng

**Affiliations:** 1 Department of Orthopaedic Surgery, National Taiwan University Hospital, Taipei City 100225, Taiwan; 2 Department of Orthopaedic Surgery, En Chu Kong Hospital, New Taipei City 237, Taiwan

**Keywords:** distal radius fracture, open reduction and internal fixation, minimally invasive, functional outcome, radiographic outcome

## Abstract

Background: The distal radius fracture is a common orthopedic injury. We aimed to share the surgical steps and investigate the outcomes of treating distal radius fractures with wounds ≤10 mm using a globally accessible locking plate. Methods: We collected 46 patients who underwent surgery via a <10 mm wound, with a control group consisting of 40 patients who underwent conventional procedures. Both groups were treated using the same volar plate. We compared the radiographic reduction quality, including volar tilt angle, radial inclination angle, and ulna variance. Additionally, clinical outcomes, such as pain assessed using VAS, Q-Dash score, and PRWE, were evaluated. Patient satisfaction with the wound was also analyzed. The follow-up time for the clinical outcomes was 24.2 ± 13.47 months. Results: There were no differences in the quality of reduction in parameters such as the volar tilt angle (*p* = 0.762), radial inclination angle (*p* = 0.986), and ulna variance (*p* = 0.166). Both groups exhibited comparable results in pain VAS (*p* = 0.684), Q-Dash score (*p* = 0.08), and PRWE (*p* = 0.134). The ≤10 mm incision group displayed an increase in satisfaction with the wound (*p* < 0.001). Conclusions: Treating distal radius fractures with a <10 mm wound using a non-specialized locking plate is a feasible approach. It does not compromise the quality of fracture reduction or functional scores and improves wound satisfaction.

## 1. Introduction

Distal radius fractures are common orthopedic injuries, frequently resulting from falls or accidents and affecting individuals of all age groups [1]. Treatment options vary based on the fracture’s severity, including casting [2,3] or surgery [4,5]. These fractures can lead to pain, reduced wrist function, and potential complications, imposing healthcare costs, impacting workforce productivity, and straining the healthcare system [6,7,8,9]. Effective diagnosis, treatment, and rehabilitation are vital to mitigate their overall impact on patients and society.

Surgical treatment for distal radius fractures offers advantages for people with higher functional demand requiring a faster recovery [10]. It enables the precise realignment of fractured bones, reducing the risk of malunion. Surgery often involves the use of internal fixation devices, such as locking plates and screws, to provide stable support during the healing process [11,12,13]. This approach is the prevailing trend in distal radius fracture surgery, maintaining alignment, enhancing stability, and facilitating early mobilization and wrist function recovery [12,13]. Locking plate systems are increasingly favored by orthopedic surgeons for their reliability and effectiveness in addressing complex fractures, leading to improved patient outcomes.

Minimally invasive surgery for distal radius fractures is currently a popular trend, typically defined by incisions smaller than three centimeters [14]. Patient satisfaction is more closely related to whether the fracture is properly aligned, while the size of the wound only affects aesthetics. However, due to advancements in surgical techniques and implant development, surgeons aspire to achieve equivalent treatment outcomes with smaller incisions, thereby minimizing the wound size to 1–1.5 cm [14,15,16,17,18]. Nonetheless, there are still some limitations, with certain studies requiring two incisions [14] or specially designed plates [14,15]. Most reports did not compare these smaller incisions to conventional techniques [14,17,18], making it challenging to assess their impact on fracture reduction and patient satisfaction regarding function and aesthetics.

This study aimed to overcome these limitations by performing distal radius fracture surgery with an ultimate small incision smaller than or equal to one centimeter. We utilized widely available locking plates instead of specially designed ones and compared the outcomes to conventional surgery. Our objectives were to assess the impact on fracture reduction, patient functional outcomes, and satisfaction with the incision. This study also details the surgical procedures, demonstrating how to perform distal radius fracture surgery with incisions smaller than or equal to one centimeter using non-specialized locking plates.

## 2. Materials and Methods

### 2.1. Patients

**Figure 1:**
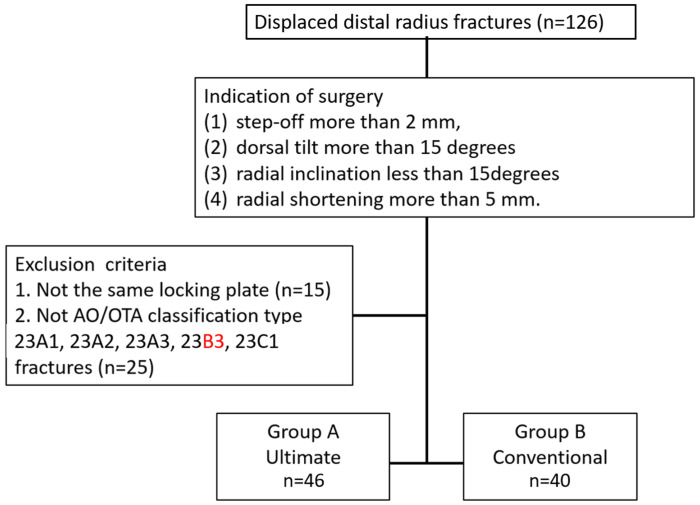
Flowchart of the study.

We conducted a retrospective cohort study in the corresponding author’s hospital. The study was approved by the hospital’s Ethics Committee, and a waiver of informed consent for the retrospective use of patient data (approval number: 202308080RIND) was obtained. We investigated 46 consecutive patients who underwent ultimate mini-incision surgery using a 2.4 mm Variable-Angle LCP Two-Column Volar Distal Radius Plate (Depuy Synthes, Oberdorf, Switzerland) for a distal radius fracture between August 2019 and January 2023. The ultimate mini-incision surgeries were all performed using THT. The indication for ultimate incision surgery was AO/OTA classification type 23A1, 23A2, 23A3, 23B3, and 23C1 adult fractures with one of the following fracture displacements: (1) step-off more than 2 mm, (2) dorsal tilt more than 15 degrees, (3) radial inclination less than 15 degrees, or (4) radial shortening more than 5 mm. Fracture types other than AO/OTA classification types 23A1, 23A2, 23A3, 23B3, and 23C1 for distal radius fractures were excluded. We collected another group of patients as the control group. These were 40 consecutive patients who underwent conventional incision surgery using the same locking plate, with the same surgical indications in the same period mentioned above. The conventional incision surgeries were performed by the co-authors, including CCL, CWC, MHH, KWW, and TMW. Figure 1 shows the flowchart of this study. The higher number of surgeons using conventional incisions despite a lower total number of patients is not indicative of these surgeons having less experience. The reason lies in the fact that in our hospital, younger surgeons have a higher frequency of on-call duties. In the years 2019–2023, THT had more on-call duties, resulting in a greater volume of fracture surgeries. On the other hand, more senior surgeons, such as those using conventional incisions, having already gone through stages with increased on-call responsibilities, contribute to this pattern. The follow-up time for clinical outcomes was 24.2 ± 13.47 months (ranging from 9 to 50 months).

### 2.2. Surgical Techniques of Ultimate Incision Surgery

**Figure 2:**
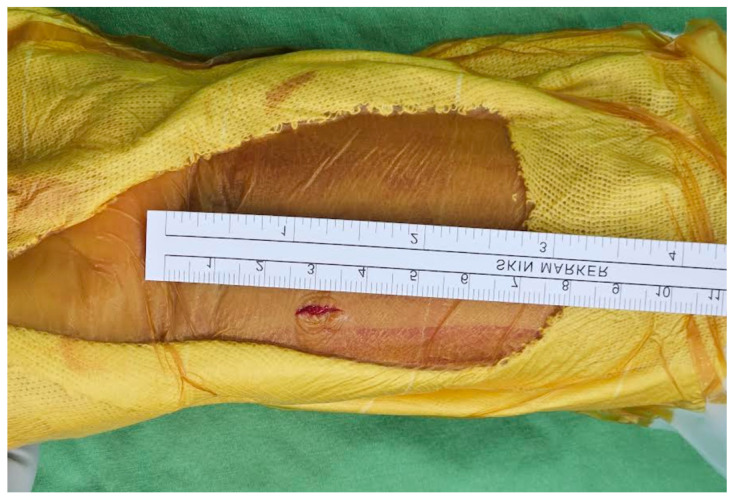
The distal end of the incision was about 25–30 mm proximal to the distal wrist crease. The length of the incision was about 8–10 mm.

**Figure 3:**
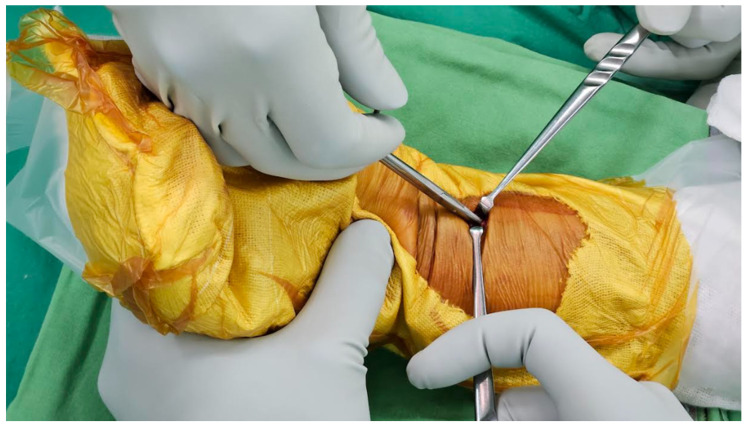
Flexion of the wrist can help increase the incised length of the proximal subcutaneous tissue and tendon sheath.

For the ultimate mini-incision surgery, the surgical technique was primarily based on a previous publication with modifications [18]. A pneumatic tourniquet is used at a pressure of 250 mmHg. A vertical incision between 8 and 10 mm was made at the radial border of the flexor carpi radialis (FCR). The distal end of the incision is about 25–30 mm proximal to the distal wrist crease (Figure 2). In general, if the pre-operative X-ray indicates that the main fracture line is within 2 cm of the joint, the surgical incision will start from the proximal 25 mm of the wrist crease. Conversely, if it is beyond 2 cm, it will start from 30 mm. The subcutaneous tissue and the superficial tendon sheath are incised using scissors as long as possible. When incising the proximal sheath, flexion of the wrist can help increase the incised length (Figure 3). After retracting the FCR tendon ulnarly, the deep tendon sheath is incised in the same manner.

In our experience, in such a small incision, deliberately preserving the pronator quadratus muscle can actually make this muscle more susceptible to injury during the surgical procedure and harder to repair. Therefore, we vertically incise the distal half of the pronator quadratus during surgery, preserving approximately 3 mm of the muscle on the radial side to facilitate subsequent repair.

**Figure 4:**
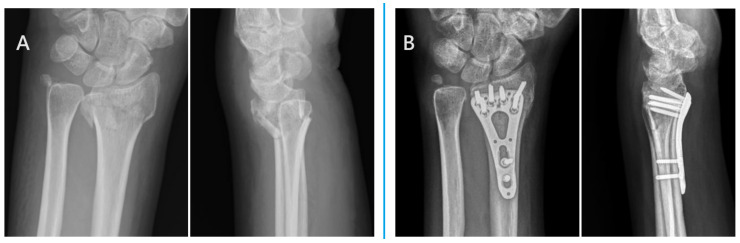
Type C1 fracture. (**A**) Pre-operative X-ray and (**B**) post-operative X-ray.

Subsequently, we begin by reducing the displaced fracture. If the volar cortex is still in contact, the fracture can typically be realigned through a simple manipulation. For instance, one finger can be inserted into the wound to support the volar cortex of the proximal fragment, while the other four fingers push the distal fragment towards the volar side to assist in reduction. Alternatively, with the fracture site as the pivot point, a bone elevator can also be used to elevate the distal fragment displaced towards the dorsal side back to its original position. In cases where there is a significant dorsal tilt, the Kapandji technique with a K-wire in the dorsal fracture may be necessary to aid in restoring volar tilt. Additionally, since we exclude comminuted joint fractures, such as type C2 and C3 fractures, and only include simple articular fractures (type C1), simply reducing the fracture line extending from the joint to the volar cortex allows joint reduction. An example of a type C1 fracture is shown in Figure 4. If managing comminuted joint fractures, the use of the ultimate incision is not recommended.

**Figure 5:**
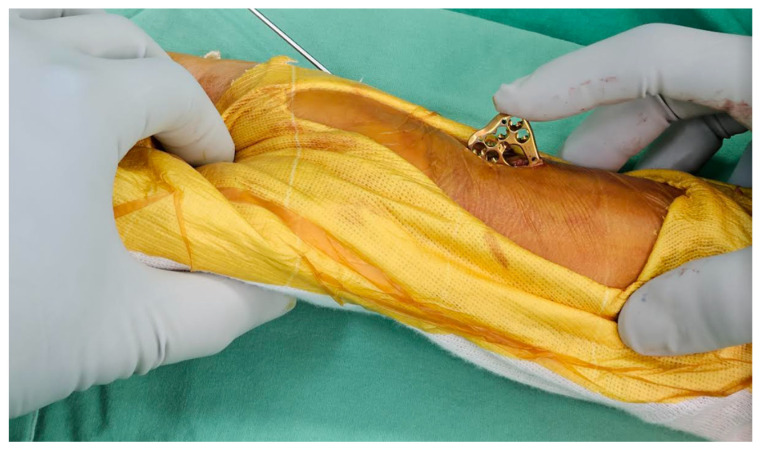
Insert the plate into the surgical wound. The plate should be oriented perpendicular to the skin, allowing one distal corner of the plate to enter the wound first.

After reducing the fracture, we typically start by temporarily stabilizing it with a K-wire, followed by inserting the locking plate into the wound. We utilize a 2.4 mm variable-angle LCP two-column volar distal radius plate (Depuy Synthes). In terms of plate length, we select a plate with two shaft holes, and the choice between the narrow design and the standard design is made based on the size of the bone. When inserting the plate, it’s essential to be mindful that the plate should be oriented perpendicular to the skin, allowing one distal corner of the plate to enter the wound first (Figure 5). Attempting to use a retractor to open the wound at this stage is not beneficial and may lead to increased wound tearing or exacerbation of the skin condition. Once the plate is inserted, it is important to ensure that the pronator quadratus is not beneath the plate. We then check the plate position with an image intensifier.

**Figure 6:**
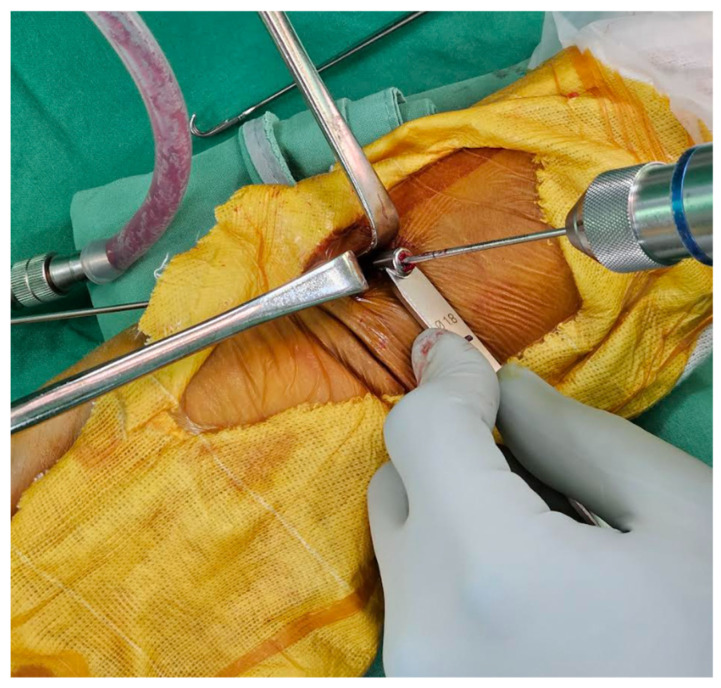
During drilling and screw insertion, it is recommended to place the retractors on the same side of the wound.

Once the correct position of the locking plate is confirmed, we will use provisional K-wires through the plate to keep it in an optimal position during the drilling and screw application. Subsequently, screws will be further inserted into the bone. Due to the smaller incision, it is recommended to place the retractors on the same side of the wound (Figure 6). Typically, within the surgical field, only the screw hole to be inserted and its immediate vicinity are visible. As long as this mobile window approach is employed, inserting distal screws should not pose any difficulty. When inserting screws near the proximal end, as mentioned earlier, it is important to maximize wrist flexion to ensure visibility of the proximal screw hole. Generally, we use six locking screws in the distal fragment and one cortical screw and one locking screw in the diaphysis.

**Figure 7:**
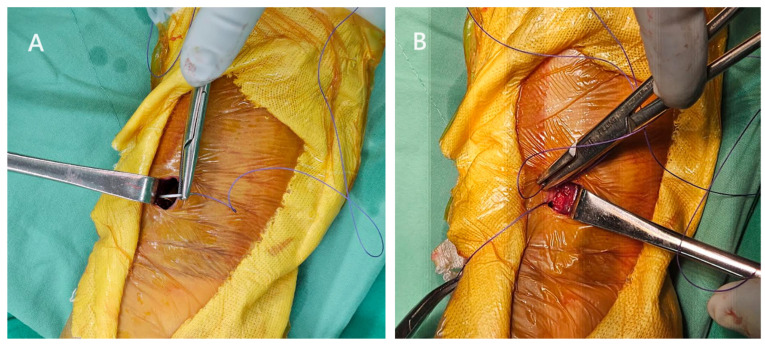
Pronator quadratus repair. (**A**) Retract the radial side where the needle was to be inserted first. (**B**) Retract the other side to suture the muscle on that side.

**Figure 8:**
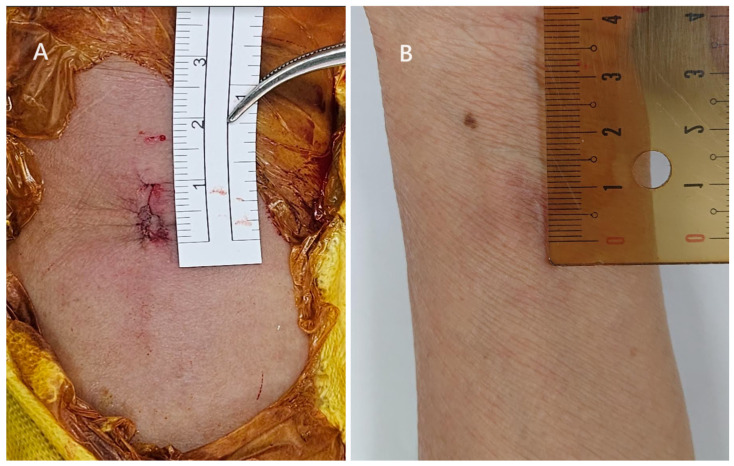
After wound closure. (**A**) Intraoperative photo. (**B**) An example of an 8 mm wound after wound healing.

When all the screws were in place, we proceeded to suture the pronator quadratus using a 2-0 Vicryl suture with a 5/8 circle needle. Due to the limited visibility, we first retracted the side where the needle was to be inserted and then retracted the other side to suture the muscle on that side (Figure 7). In cases of significant displacement of the fracture, the pronator quadratus is often partially injured. Therefore, under normal circumstances, we were able to suture more than 2/3 of the dissected muscle, but suturing the most distal portion of the muscle was more challenging. The wound is finally closed with subcutaneous sutures using 3-0 Vicryl followed by 4-0 Vicryl in sequence (Figure 8). After the surgery, we instruct patients to wear a splint for two weeks for protection. After the two-week period, patients are no longer required to wear the splint, and we encourage them to start gentle wrist exercises.

### 2.3. Surgical Techniques of Conventional Incision Surgery

A pneumatic tourniquet is used at a pressure of 250 mmHg. A vertical incision between 30 and 50 mm is made at the radial border of the FCR. The distal end of the incision is about 15 mm proximal to the distal wrist crease. The subcutaneous tissue and the superficial tendon sheath were incised using scissors. After retracting the FCR tendon ulnarly, the deep tendon sheath was incised in the same manner. The pronator quadratus was incised vertically, preserving approximately 3 mm of the muscle on the radial side to facilitate subsequent repair. After the displaced fracture is reduced by manipulation and/or the Kapandji technique, a K-wire is often used through the radial styloid to temporarily stabilize it. A locking plate is then applied, and we check the plate position with an image intensifier. Screws are inserted for the final fixation of the fracture. The pronator quadratus is repaired using 2-0 Vicryl. The wound is finally closed with subcutaneous sutures using 3-0 Vicryl followed by 4-0 Vicryl in sequence. After the surgery, we instruct patients to wear a splint for two weeks for protection. After the two-week period, patients are no longer required to wear the splint, and we encourage them to start gentle wrist exercises.

### 2.4. Outcome Evaluations

The radiographic outcome, which included measurements of the volar tilt angle, radial inclination angle, ulna variance, and Soong grade [19] of the plate position, was assessed based on immediate post-operative plain films to determine the extent of fracture reduction. After the surgery, we conducted the same monthly X-ray imaging examination for the first three months post-surgery and approximately the sixth month to determine if there was any subsequent displacement. Taking into account measurement errors, we defined an angle change greater than 3 degrees and a distance measurement greater than 3 mm as indicative of subsequent displacement. All radiographic parameters were measured by CYH and THT in a blinded manner. Both observers measured the parameters twice, and the intervals between each time were >4 weeks.

We assessed patients’ clinical outcomes in Oct 2023 using the visual analog scale (VAS) for pain intensity and patient-reported outcome measures, including the quick disabilities of the arm, shoulder, and hand (Q-DASH) score and patient-rated wrist evaluation (PRWE) [20]. Patient wound satisfaction was evaluated using a numerical rating scale (NRS), where patients provided a subjective score corresponding to their level of satisfaction, with 0 indicating the highest satisfaction and 10 indicating the lowest satisfaction. We also analyzed the complications in both groups, including those documented in the medical records and subsequent displacement of the fractures.

### 2.5. Statistical Analysis

All statistical analyses were performed using Real Statistics Resource Pack software (release 8.0) on a Microsoft Windows-based computer. The chi-square test was used to determine if two categorical variables between two groups are independent or if they are, in fact, related to one another. The Mann–Whitney U test was employed to compare the difference of continuous data between the two groups. Statistical significance was set at a *p*-value of <0.05. The interobserver and intraobserver reliabilities of the radiographic parameters were assessed using the intraclass correlation coefficient (ICC; model: two-way random; type: absolute agreement, single measures).

## 3. Results

### 3.1. Patient Characteristics

The characteristics of the patients are listed in Table 1. There were no significant differences among patients in terms of age, gender, BMI, injured side, and fracture classification. All cases in which an ultimate incision was intended result in an ultimate incision.

### 3.2. Radiographic Outcome

Regarding immediate post-operative radiographic outcome, there were no differences in the quality of fracture reduction in various parameters such as volar tilt angle, radial inclination angle, and ulna variance (Table 2). The mean time to union was 2.82 ± 0.4 months in the ultimate group and 2.68 ± 0.4 months in the conventional group (*p* = 0.784). The interobserver reliability (95% confidence interval of ICC: pre-operative volar tilt: 0.90–0.94, 0.89–0.93; radial inclination: 0.91–0.96, 0.90–0.95; ulna variance: 0.91–0.96, 0.88–0.94. Post-operative volar tilt: 0.90–0.94, 0.90–0.96; radial inclination: 0.89–0.94, 0.91–0.96; ulna variance: 0.88–0.94, 0.91–0.95 for each repeat) and intraobserver reliability (95% confidence interval of ICC: pre-operative volar tilt: 0.92–0.96, 0.90–0.94; radial inclination: 0.89–0.94, 0.91–0.96; ulna variance: 0.92–0.96, 0.90–0.95. Post-operative volar tilt: 0.91–0.96, 0.89–0.94; radial inclination: 0.89–0.96, 0.90–0.94; ulna variance: 0.88–0.95, 0.90–0.95 for each observer) were all high.

### 3.3. Clinical Outcome

As for the clinical outcomes, both groups exhibited comparable results in the VAS, Q-Dash score, and PRWE. However, the ultimate mini-incision group displayed a significant increase in patient satisfaction with the wound, and this group also demonstrated a significantly shorter surgical duration compared to the other group (Table 3). The difference in surgical duration in minutes was 19.5 min.

### 3.4. Complications

In the ultimate mini-incision group, two cases experienced fracture re-displacement, while in the other group, one patient had an extensor pollicis longus rupture, and two had fracture re-displacement. Since all five patients were unwilling to undergo surgery again, conservative treatment was applied for these complications.

## 4. Discussion

The primary contribution of this study lies in confirming that, with the appropriate surgical indications, utilizing incisions smaller than one centimeter can yield clinical results and reduction quality comparable to conventional minimally invasive techniques. Moreover, there is a significant enhancement in patient satisfaction regarding the incision. Furthermore, the use of globally accessible locking plates in the surgery, as opposed to specially designed plates, provides surgeons reading this article with increased confidence in adopting this minimally invasive surgical approach by following our outlined procedural steps. The pearls and pitfalls of the technique are listed in Table 4.

Many studies have explored the reduction of surgical incisions in the treatment of distal radius fractures. For example, Asmar et al. achieved open reduction and internal fixation of distal radius fractures through a 32 mm incision using a newly designed ultra-short plate, resulting in favorable clinical outcomes within a short surgical duration [21]. Other research endeavors have focused on further minimizing incision sizes. Lebailly et al., for instance, successfully utilized a volar plate (Step One^®^, Newclip Technics™, Haute-Goulaine, France) through a 15 mm incision, demonstrating its feasibility [21]. Subsequent investigations took a step further in reducing incision sizes. Ribeiro et al. reduced the incision to 1.2 cm, albeit necessitating two transverse incisions and the use of a specially designed plate [14]. Naito et al. reported a case series confirming that a 1 cm incision enables satisfactory functional recovery in patients [18], though the absence of a control group raises uncertainty about whether such small incisions might compromise clinical function and reduction quality. The present study builds upon these encouraging findings, aiming to extend the application of treating distal radius fractures with incisions smaller than or equal to one centimeter, utilizing a non-specially designed locking plate. We demonstrate that comparable quality of reduction and clinical outcomes can be achieved compared to larger incisions. Our results also indicate that the ultimate incisions significantly enhance patient satisfaction with the wound appearance. In our experience, patients are often surprised and impressed by the placement of the plate through such small incisions, boosting confidence in the surgeon’s skill, which may be reflected in their satisfaction with the wound. Another unexpected finding is a significant reduction in surgical time. We attribute this to the smaller incisions and decreased soft tissue dissection, leading to a shorter duration for wound closure. Another potential contributing factor is the difference in surgeons; the diverse experiences and procedural rhythms among the surgical practitioners in the two groups may inevitably vary, possibly leading to the observed outcome.

Given that the pronator quadratus functions as a secondary forearm pronator and dynamically stabilizes the distal radioulnar joint, and an intact pronator quadratus can serve as a biological barrier for the flexor tendons, the question of whether the integrity or repair of the pronator quadratus contributes to clinical outcomes is a frequently debated topic [22,23,24]. Despite diverse perspectives in the literature [25,26,27,28], current high-level evidence suggests that the integrity or repair of the pronator quadratus does not definitively impact clinical results [22,23,24,29]. In our clinical practice, displaced fractures of the distal radius often coincide with a partial tear of the pronator quadratus. Attempting to preserve the pronator quadratus during surgery may also result in muscle injury, leading us to partially release the muscle during the procedure. Although there is no conclusive evidence mandating pronator quadratus repair, we have been using a mobile surgical window approach to repair the majority of the released muscle during surgery. Current follow-up results indicate no issues with flexor tendons, and patients demonstrate satisfactory functional scores. Another potential factor contributing to flexor tendon issues is the positioning of the locking plate. The reported rate of flexor tendon rupture after volar plates is around 0.57% [30,31,32,33], and some studies indicate that volar locking plate prominence is a risk factor for flexor tendon rupture, especially in Soong grade 2 cases [34]. Therefore, we made an effort during surgery to avoid unnecessarily placing the locking plate in Soong grade 2 conditions. This may be one of the reasons why there are currently no issues with flexor tendons, but long-term follow-up is necessary to confirm this.

**Figure 9:**
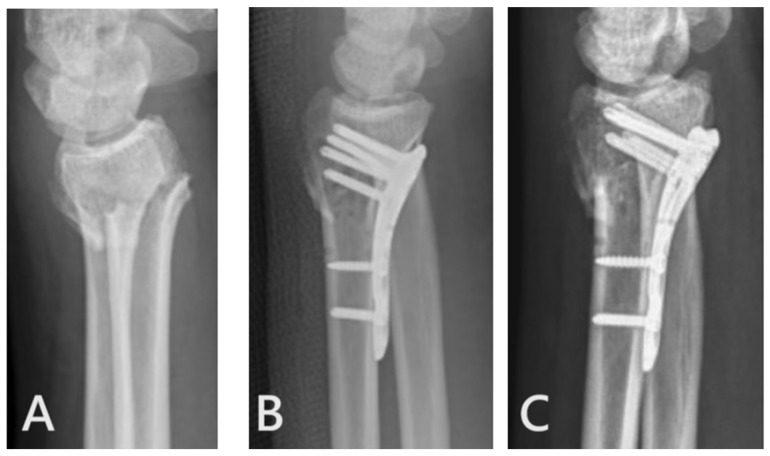
Re-displacement of fracture. (**A**) Pre-operative X-ray. (**B**) Immediate post-operative X-ray. (**C**) Follow-up X-ray.

In the “ultimate incision” group, there were two instances of post-operative re-displacement observed. We attribute this to the suboptimal placement of the locking plate. Taking one patient with a re-displaced fracture as an example, the plate was positioned too proximally, resulting in insufficient fixation strength of the distal screw on the distal bone fragment and subsequent re-displacement (Figure 9). Due to the constrained visibility in small incisions, there is a tendency for the plate to be situated in less-than-ideal positions, leading to fracture re-displacement. As a precautionary measure, we recommend placing the plate and inserting the initial 2–3 screws, followed by reassessing the plate position using an image intensifier. Once the position is confirmed to be satisfactory, the remaining screws can then be inserted to minimize the risk of fracture re-displacement.

This study has several limitations. Firstly, the patient follow-up duration is not extensive, with the shortest follow-up period being nine months. However, this duration is sufficient to assess reduction quality and alignment after the union of distal radius fractures. Both patient groups underwent surgery during the same period, making the comparison of clinical outcomes between them representative, although long-term follow-up remains essential. Secondly, in our view, the “ultimate incision” approach may not be suitable for cases with comminuted joint surfaces. For joint fractures, we only included fractures with a C1 fracture pattern, as they usually behave like extraarticular fractures and can be well-managed even with the “ultimate incision” approach. Therefore, when using this surgical method, selecting appropriate surgical indications is crucial. Thirdly, as this is a retrospective study, there is still a possibility of selection bias in the fracture patterns of the patients. Future prospective studies are warranted to obtain a more thorough comparison in this aspect. Fourthly, because this study is a retrospective investigation and the number of patients is limited, although no serious complications occurred among these patients, it is not possible to confirm the potential for severe complications through this study. This needs to be verified through future larger-scale studies. Lastly, the ultimate incision group was all operated on by THT. Prior to performing surgery on this group of patients, the surgeon dedicated two years to surgeries on approximately 30 patients. During this period, the incision size progressed from 2 cm to 1.5 cm and was further reduced to 1 cm. Therefore, this surgical procedure entails a learning curve. The hope is that sharing insights from this study will help minimize the learning curve for other surgeons.

## 5. Conclusions

This study confirmed that treating distal radius fractures with wounds smaller than 10 mm using a non-specialized locking plate is a viable therapeutic approach. With regard to short-term outcomes, it does not compromise the quality of fracture reduction or functional scores and significantly improves wound satisfaction.

## Figures and Tables

**Table 1:** Patient characteristics.

	Conventional	Ultimate Incision	*p*-Value
Number of patients	40	46	
Age, mean ± standard deviation (SD)	63.4 ± 16.0	60.7 ± 15.2	0.530
Sex (male/female)	12/28	8/38	0.167
Body mass index (BMI)	27.6 ± 2.1	26.5 ± 2.3	0.684
Laterality (right/left)	19/21	19/27	0.564
AO/OTA classification (A/B3/C1)	27/4/9	38/1/7	0.099

**Table 2:** Radiographic outcome.

	Conventional	Ultimate Incision	*p*-Value
Pre-operative parameter (mean ± SD)			
Volar tilt angle (°)	−13.1 ± 16.3	−14.6 ± 18.6	0.652
Radial inclination angle (°)	13.8 ± 9.8	15.0 ± 9.6	0.774
Ulna variance (mm)	3.1 ± 1.3	2.9 ± 1.5	0.446
Immediate post-operative parameter (mean ± SD)			
Volar tilt angle (°)	9.6 ± 5.0	9.0 ± 5.2	0.762
Radial inclination angle (°)	21.7 ± 4.2	21.9 ± 2.9	0.986
Ulna variance (mm)	0.5 ± 1.2	0.9 ± 1.2	0.166
Soong grade (grade 0/1/2)	20/18/2	29/14/3	0.378

**Table 3:** Clinical outcome.

Parameters (Mean ± SD)	Conventional	Ultimate Incision	*p*-Value
Pain VAS	0.7 ± 0.8	0.6 ± 0.7	0.684
Q-DASH	9.26 ± 10.6	5.42 ± 7.67	0.080
PRWE	12.2 ± 4.3	10.3 ± 4.1	0.134
Cosmetic NRS	1.93 ± 1.57	0.68 ± 0.87	<0.001
Surgical duration (min)	76.3 ± 22.4	59.8 ± 12.6	<0.001

The surgical duration is defined as the time from the first incision to the last suture.

**Table 4:** Pearls and pitfalls.

	Pearls	Pitfalls
Fracture type	Metaphysis and/or simple articular fractures	Avoid comminuted articular fractures.
Fracture reduction	Finger manipulation for fractures with volar cortices in contact; Kapandji technique for large dorsal tilt; Use of a bone levator, with the fracture site as the pivot point, to reduce the distal fragment;	Difficult to insert traditional reduction forceps into the ultimate incision.
Plate insertion	The plate is perpendicular to the skin, allowing one distal corner of the plate to enter the wound first; Ensure that the pronator quadratus is not beneath the plate;	Avoid using retractors when inserting the plate.
Screw insertion	Mobile window approach; Wrist flexion for proximal screws.	Avoid forceful wound retraction.

## Data Availability

The data that support the findings of this study are available from the corresponding author upon reasonable request.
